# Phase II study of everolimus and temozolomide as first-line treatment in metastatic high-grade gastroenteropancreatic neuroendocrine neoplasms

**DOI:** 10.1038/s41416-023-02462-0

**Published:** 2023-10-23

**Authors:** Siren Morken, Seppo W. Langer, Anna Sundlöv, Lene Weber Vestermark, Morten Ladekarl, Geir Olav Hjortland, Johanna B. Svensson, Elizaveta Mitkina Tabaksblat, Torjan Magne Haslerud, Jörg Assmus, Sönke Detlefsen, Anne Couvelard, Aurel Perren, Halfdan Sorbye

**Affiliations:** 1 Department of Oncology, Haukeland University Hospital, Bergen, Norway; 2 Department of Oncology, Rigshospitalet, Copenhagen, Denmark; 3 Department of Clinical Medicine, University of Copenhagen, Copenhagen, Denmark; 4 Division of Oncology, Department of Clinical Sciences Lund, Lund University, Lund, Sweden; 5 Department of Oncology, Odense University Hospital, Odense, Denmark; 6 Department of Oncology, Aarhus University Hospital, Aarhus, Denmark; 7 Department of Oncology, Clinical Cancer Research Center, Aalborg University Hospital, Aalborg, Denmark; 8 Department of Clinical Medicine, Aalborg University, Aalborg, Denmark; 9 Department of Oncology, Oslo University Hospital, Oslo, Norway; 10 Department of Oncology, Sahlgrenska University Hospital, Gothenburg, Sweden; 11 Department of Radiology and Nuclear Medicine, Haukeland University Hospital, Bergen, Norway; 12 Centre for Clinical Research, Haukeland University Hospital, Bergen, Norway; 13 Department of Pathology, Odense University Hospital, Odense, Denmark; 14 Department of Clinical Research, Faculty of Health Sciences, University of Southern Denmark, Odense, Denmark; 15 Department of Pathology, Bichat Hospital, Paris, France; 16 Institute of Tissue medicine and Pathology, University of Bern, Bern, Switzerland; 17 Department of Clinical Science, University of Bergen, Bergen, Norway

**Keywords:** Neuroendocrine cancer, Phase II trials

## Abstract

**Background:**

The optimal treatment for metastatic high-grade gastroenteropancreatic (GEP) neuroendocrine neoplasms when Ki-67 ≤55% is unknown. A prospective multi-centre phase 2 study was performed to evaluate the efficacy and safety of everolimus and temozolomide as first-line treatment for these patients.

**Methods:**

Patients received everolimus 10 mg daily continuously and temozolomide 150 mg/m^2^ for 7 days every 2 weeks. Endpoints included response, survival, safety and quality of life (QoL). Histopathological re-evaluation according to the 2019 WHO classification was performed.

**Results:**

For 37 eligible patients, the primary endpoint with 65% disease control rate (DCR) at 6 months (m) was reached. The response rate was 30%, the median progression-free survival (PFS) 10.2 months and the median overall survival (OS) 26.4 months. Considering 26 NET G3 patients, 6 months DCR was 77% vs. 22% among nine NEC patients (*p* = 0.006). PFS was superior for NET G3 vs. NEC (12.6 months vs. 3.4 months, Log-rank-test: *p* = 0.133, Breslow-test: *p* < 0.001). OS was significantly better for NET G3 (31.4 months vs. 7.8 months, *p* = 0.003). Grade 3 and 4 toxicities were reported in 43% and 38%. QoL remained stable during treatment.

**Conclusion:**

Everolimus and temozolomide may be a treatment option for selected GEP-NET G3 patients including careful monitoring. Toxicity did not compromise QoL.

**Clinical trial registration:**

ClinicalTrials.gov (NTC02248012).

## Background

Gastroenteropancreatic neuroendocrine neoplasms (GEP-NEN) are a heterogeneous group of neoplasms differing in biological behaviour, treatment benefit, and survival. The 2019 WHO classification of tumours of the digestive system separates high-grade (HG) GEP-NEN with a Ki-67 index above 20% into two distinct entities; well-differentiated neuroendocrine tumours, NET G3, and poorly differentiated neuroendocrine carcinoma, NEC [1]. Despite being a rare disease, the incidence of HG GEP-NEN is increasing [2]. Most HG GEP-NEN patients have metastases at the time of diagnosis (60-78%) [3–6]. GEP-NEC has an aggressive disease course, while GEP-NET G3 has a prognosis intermediate between NET G2 and NEC. In previous studies, median overall survival (OS) of patients with metastatic GEP-NET G3 has been reported around 31–42 months (m) [7–9] as compared to 11–12 months in metastatic GEP-NEC patients receiving palliative chemotherapy [6, 10–12].

Traditionally patients with both GEP-NET G3 and GEP-NEC have predominantly received platinum-based chemotherapy as first-line palliative treatment [13, 14]. The Nordic NEC study showed that HG GEP-NEN with a Ki-67 < 55% had a lower response rate (RR) to platinum/etoposide compared to patients with Ki-67 > 55% (15 vs. 42%) [13]. Moreover, newer data show that platinum/etoposide is not an optimal treatment for NET G3 [6]. Therefore, guidelines generally advocate first-line palliative treatment with platinum/etoposide for metastatic GEP-NEC [15–18], whereas the optimal treatment for GEP-NET G3 and NEC with Ki-67 ≤ 55% remains uncertain [19, 20]. Both everolimus and temozolomide-based chemotherapy are established treatments for GEP-NET G1-2 [21, 22]. The combination of everolimus and temozolomide was shown safe and effective in a prospective study of advanced pancreatic NET G1-2 [23]. Recent studies have shown encouraging results for both temozolomide-based chemotherapy and everolimus in GEP-NET G3 [7–9, 24]. Temozolomide-based chemotherapy has shown promising results for HG GEP-NEN in a second-line setting, especially when Ki-67 < 55% [25], as well as for GEP-NEC [15–18]. Current guidelines frequently suggest first-line treatment with a temozolomide-based regimen for metastatic GEP-NET G3 [15, 16, 18], with an exception for aggressive GEP-NET G3 cases (e.g., with Ki-67 ≥ 55%) [17]. Guidelines include a temozolomide-based regime as a treatment option for metastatic GEP-NEC [15–18].

Recognising the unmet need for clinical trial data to improve the treatment of advanced HG GEP-NEN with Ki-67 21–55% and demonstrated efficacy of both everolimus and temozolomide in GEP-NET G1-2, this study was conducted to evaluate the efficacy and safety of the combination of everolimus and temozolomide as a first-line treatment for this patient group. Pathological reclassification was performed to separate NET G3 and NEC patients according to the WHO 2019 classification of tumours of the digestive system [1].

## Methods

### Study design and patients

This study was a non-randomised, single-arm, first-line prospective multi-centre phase II trial. Patients were included with histologically confirmed and advanced high-grade neuroendocrine neoplasms of gastroenteropancreatic or of unknown origin with a predominance of gastrointestinal metastasis. The study had the following inclusion criteria: WHO performance status of 0–1, tumour Ki-67 index of 21–55%, no previous therapy for advanced/metastatic disease, and measurable disease according to Response Evaluation Criteria in Solid Tumours version 1.1 (RECIST v. 1.1).

### Treatment and evaluation

Everolimus 10 mg daily continuously was combined with temozolomide 150 mg/m^2^ for 7 days every 2 weeks. One treatment cycle was defined as 28 days. Temozolomide has been shown to be an active agent in HG GEP-NEN with a standard 4-week regime [25]. In this study, the intention was to increase efficacy further by using a dose intensive 2-week regime and adding everolimus. The dosing schedule for everolimus and temozolomide was selected from a prior study on pancreatic NET, where this schedule was found safe, efficacious and with good tolerability [23]. When treatment continued for >2 months, patients received pneumocystis jirovecii prophylaxis with trimethoprim-sulfamethoxazole. Treatment continued until death, disease progression, unacceptable toxicity, or withdrawal of consent, whichever came first.

For haematological toxicities, both drugs were held at the occurrence of Grade ≥3 toxicity and re-administered at the subordinate dose level (everolimus 5 mg daily continuously and temozolomide 100 mg/m^2^ 7 days on and 7 days off) when recovery to Grade ≤2 toxicity. For non-haematological toxicities (excluding metabolic events, pneumonitis and stomatitis), the occurrence of Grade ≥3 toxicity led to temporary dose interruption of both drugs until recovery to Grade ≤1 toxicity and then reintroduction of both drugs at a lower dose. Grade 4 toxicity led to discontinuation of both drugs. In the case of metabolic events, Grade ≥3 toxicity led to dose interruption of everolimus and re-initiation of everolimus at a lower dose. Grade 4 toxicity led to the discontinuation of everolimus. Treatment with both drugs was suspended in the event of Grade ≥2 pneumonitis and stomatitis and re-administered at recovery to Grade ≤1 toxicity to the subordinate dose level for pneumonitis and with an unaltered dose for stomatitis.

CT evaluation was performed at baseline (before 4 weeks of treatment initiation), after 6 weeks of treatment, and thereafter every 8 weeks, and the response was evaluated according to RECIST v. 1.1. [18F]FDG-PET/CT was performed at baseline and after 6 weeks of treatment with response evaluation according to PET Response Criteria in Solid Tumours version 1.0 (PERCIST v. 1.0) [26]. To normalise the tumour SUV_max_ values, the tumour-to-liver SUV_max_ uptake ratio (SUV_TLR_) was calculated.

### Re-evaluation of pathology

At the time of protocol development (2014) and study enrolment (2014-2017), all GEP-NEN with a Ki-67 > 20% were classified as neuroendocrine carcinoma, NEC G3. The 2019 WHO pathology grading system divides HG GEP-NEN into the well-differentiated NET G3 and the poorly differentiated NEC [1]. As a result of the new classification, all patients were blinded and re-evaluated in 2021 according to morphology, immunohistochemically staining (synaptophysin and chromogranin A) and Ki-67 index, simultaneously and individually by two experienced NEN pathologists (A.P. and A.C.). Conflicting or uncertain cases were discussed among them to form a consensus. All HG-NEN patients were included in the final analysis, including cases with a Ki-67 > 55% after re-evaluation.

### Outcomes

An interim analysis was planned to evaluate the toxicity and benefit of treatment for the first 20 consecutive patients. If >50% of the patients experienced dose-limiting toxicity, a trial amendment with revised study drug dosing would be performed. The study would be terminated for lack of efficacy if <60% had at best non-progressive disease after 6 weeks of treatment.

The primary endpoint was disease control rate (DCR) at 6 months, equivalent to non-progressive disease at 6 months according to RECIST version 1.1. In the Nordic NEC study, DCR at 6 months was 38% for a similar patient population (Ki-67 20–55% and PS 0–1) and the use of temozolomide-based chemotherapy as second-line treatment in GEP-NEC resulted in a general disease control rate of 72% at 2 months, and was even higher if Ki-67 was <60% [25]. With the use of everolimus and temozolomide as first-line treatment, an increase in DCR at 6 months from 38% to 58% was expected. Using *α* = 0.05 and power 0.80, the required sample size was calculated to 37 evaluable patients. Sample size estimation was done by Fleming’s single-stage procedure for phase II trials. Secondary endpoints included OS, PFS, RR, duration of response (DOR), safety profile according to Common Terminology Criteria for Adverse Events (CTCAE) version 4.0 and quality of life (QoL). OS was assessed from the date of the first study treatment to the date of death or last follow-up. There was a pre-planned subgroup analysis on objective tumour response, PFS, and OS according to [18F]FDG-PET/CT uptake. Metabolic tumour response was defined by PERCIST v. 1.0. Exploratory objectives included studies of predictive and prognostic factors. Subgroup analyses were performed for NET G3 and NEC according to primary and secondary endpoints, as well as 3- and 5-year survival.

### Quality of life

Quality of life during the trial was evaluated using the European Organisation for Research and Treatment of Cancer QoL questionnaire (EORTC QLQ-C30). The QoL questionnaire was to be completed at baseline, 6 weeks after the start of treatment, and then every 8 weeks during the entire treatment course. The 30-item questionnaire included a global health status/QoL scale, five functional scales (physical, role, emotional, cognitive, and social functioning), three symptom scales (fatigue, nausea/vomiting, pain), and single items assessing additional symptoms (dyspnoea, insomnia, appetite loss, constipation, diarrhoea) and financial difficulties. The results were linearly transformed into a score from 0–100 according to the EORTC scoring manual. For the QoL and functioning scales, a higher score represented a better outcome. For the symptom scales, a high score represented an increased burden of symptoms.

### Statistics

Patient and tumour characteristics were analysed using descriptive statistics. Continuous variables (DOR, PFS and OS) were analysed using the Kaplan–Meier method, log-rank test, and Breslow-test. Median follow-up time was analysed with the use of the reverse Kaplan–Meier method. Univariate analyses were performed using Cox regression. Multivariate analyses were not performed due to the limited number of patients. Subgroup analyses were performed for NET G3 and NEC. Comparisons of DCR at 6 months for NET G3 and NEC were assessed by the Exact Chi-square test. A *p*-value of less than 0.05 was considered significant. The sample size estimation was done by Fleming single-stage procedure, and the target accrual was 37 patients. Statistical analyses were performed using IBM SPSS statistics version 26.0. Figures were created using MatLab 9.0.

## Results

The assessment of adverse event rates for the first 20 patients showed that 75% needed dose reduction and 65% needed dose delay, but only three patients discontinued treatment due to toxicity. The interim analysis demonstrated a DCR of 90% at 6 weeks. Therefore, the study proceeded without adjustments to the study medication. From November 2014 to December 2017, 38 patients were recruited from seven centres in Denmark, Sweden, and Norway. One participant was excluded from further analysis during the enrolment period due to additional pathological information redefining the case as having non-NEN disease. The cut-off date for follow-up was August 17, 2021, where nine of 37 patients were still alive.

### Patient and tumour characteristics

Baseline characteristics for the 37 patients included are presented in Table 1. The median age was 66 years (range 33-79), most patients had metastatic disease (97%) and the most common primary tumour site was the pancreas (38%). Somatostatin receptor imaging (SRI) was positive in 21/28 patients (75%). Thirty-three patients had [18F]FDG-PET/CT performed at baseline, all showed FDG-PET uptake. *TSC1/2, MTOR*, or *PTEN* mutations, which may indicate response to everolimus, were not found among the nine cases with an NGS panel available.

**Table 1:** Baseline patient and tumour characteristics.

	Valid cases	All cases	NET G3	NEC
Total number	37	37	26 (70%)	9 (24%)
Age, median (range)	37	66 (33-79)	66 (41–79)	67 (33–74)
PS (WHO)	37			
0		17 (46%)	12 (46%)	3 (33%)
1		20 (54%)	14 (54%)	6 (67%)
Gender	37			
Male		20 (54%)	14 (54%)	5 (56%)
Female		17 (46%)	12 (46%)	4 (44%)
Primary tumour site	37			
Pancreas		14 (38%)	11 (42%)	3 (34%)
Oesophagus		2 (5%)		1 (11%)
Gastric		3 (8%)	1 (4%)	2 (22%)
Gallbladder/duct		1 (3%)	1 (4%)	
Small bowel		5 (13%)	5 (19%)	
Colon left		1 (3%)		1 (11%)
Rectum		3 (8%)	2 (8%)	1 (11%)
Unknown^a^		8 (22%)	6 (23%)	1 (11%)
Primary tumour resected	37	4 (11%)	2 (8%)	2 (22%)
Metastatic disease	37	36 (97%)	25 (96%)	9 (100%)
Ki-67 index, median (range)^b^	37	38 (21–90)	29 (21–80)	50 (40–90)
Ki-67 21–55%		30 (81%)	24 (92%)	5 (56%)
Ki-67 > 55%		7 (19%)	2 (8%)	4 (44%)
Positive [18 F]FDG-PET^c^	33	33 (100%)	24 (100%)	7 (100%)
SRI uptake >liver^c,d^	28	21 (75%)	17 (85%)	3 (43%)
CgA staining^c^	35			
Strongly positive		29 (83%)	20 (83%)	7 (78%)
Partly positive		6 (17%)	4 (17%)	2 (22%)
Synaptophysin staining	37			
Strongly positive		34 (92%)	23 (89%)	9 (100%)
Partly positive		3 (8%)	3 (11%)	
CgA serum level > UNL^c^	34	27 (79%)	21 (91%)	4 (44%)
LDH >UNL^c^	30	13 (43%)	7 (35%)	5 (63%)
ALP >UNL^c^	33	14 (42%)	9 (41%)	3 (33%)
Platelets >400 × 10^9^/L^c^	34	1 (3%)	1 (4%)	
CRP > 10 mg/L	37	12 (32%)	8 (31%)	3 (33%)

*NET* neuroendocrine tumour, *NEC* neuroendocrine carcinoma, *PS* performance status, [18F]FDG-PET fluorodeoxyglucose-positron emission tomography, *SRI* somatostatin receptor imaging, *CgA* chromogranin A, *UNL* upper normal limit, *LDH* lactate dehydrogenase, *ALP* alkaline phosphatase, *CRP* C-reactive protein.

^a^Unkown primary with the dominance of gastrointestinal metastases.

^b^Reassessed.

^c^Percentage as a fraction of examined patients.

^d^68Ga-DOTATATE PET/CT (*n* = 9), 111In-octreotide SPECT (*n* = 27), both imaging modalities (*n* = 7, with same result).

### Efficacy

**Fig. 1:**
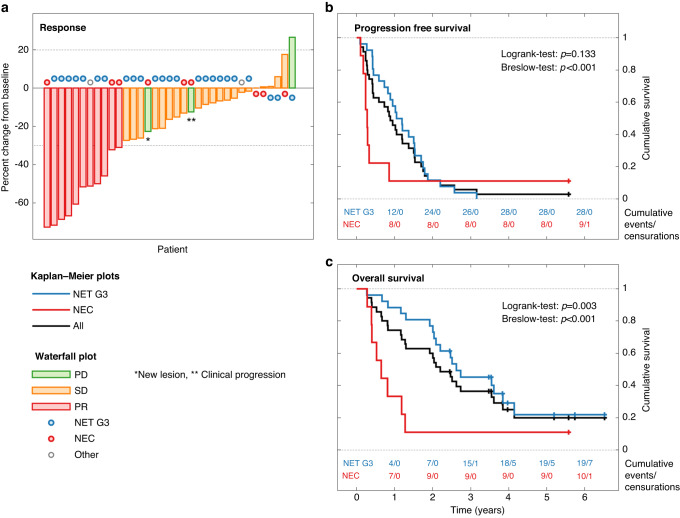
Response and survival in 37 HG GEP-NEN patients receiving first-line treatment with everolimus/temozolomide. Waterfall-plot (**a**) showing treatment response for all patients, the maximum change in the sum of the target lesion. Kaplan–Meier curves showing median progression-free (**b**) and median overall (**c**) survival for all patients and for the NET G3 and NEC sub-groups.

With a median follow-up period of 62 months, the median number of treatment cycles was nine (range 1–34), and the median duration of treatment was 8.6 months (range 0.7–31.0 months) (Table 2). The primary endpoint was achieved, as DCR at 6 months was 65% in the whole cohort; three patients (8%) experienced immediate PD (two radiological and one only clinical PD). Eleven of 37 patients (30%) experienced a partial response (PR) with a median DOR of 13.0 months (range 1.4–63.2 months), and another 23 patients achieved stable disease (SD) (Fig. 1a). PFS and OS for the 37 patients were 10.2 months (95% CI: 6.4–14.0 months) and OS was 26.4 months (95% CI: 19.8–33.0 months), respectively (Fig. 1b, c). Most patients (68%) discontinued treatment due to progressive disease, five patients (14%) due to treatment-related toxicity, and another five due to patient request. One patient discontinued treatment after study finalisation after 2.3 years of treatment (without progression). Estimated 3-year survival was 32%. Four patients (11%) had survival beyond 5 years, all with PR to first-line treatment.

**Table 2:** Treatment, response and survival.

	Valid cases	All cases	NET G3	NEC	*p*-value
Total number	37	37	26	9	
Treatment cycles, median (range)	37	9.0 (1–34)	13.5 (1–34)	3.0 (2–10)	
Treatment duration, median^a^ (range)	37	8.6 (0.7–31.0)	12.3 (0.7–31.0)	2.4 (0.9–9.0)	
Overall best response	37				
PR		11 (30%)	7 (27%)	3 (33%)	
SD		23 (62%)	18 (69%)	4 (44%)	
PD		3 (8%)	1 (4%)	2 (22%)	
mDOR (range)^a^	11	13.0 (1.4–63.2)	13.2 (6.9–23.0)	1.5 (1.4–63.2)	0.848
DCR	37	34 (92%)	25 (96%)	7 (78%)	
DCR 6 months	37	24 (65%)	20 (77%)	2 (22%)	0.006
mPFS (95% CI)^a^	37	10.2 (6.4–14.0)	12.6 (8.7–16.5)	3.4 (2.0–4.7)	0.133 <0.001^c^
mOS (95% CI)^a^	37	26.4 (19.8–33.0)	31.4 (17.0–45.9)	7.8 (3.2–12.4)	0.003 <0.001^c^
Three-year survival	37	12 (32%)	11 (42%)	1 (11%)	
Second-line treatment	37	28 (76%)	21 (81%)	6 (67%)	
Type of second-line treatment	28				
Carboplatin/etoposide		9 (32%)	4 (19%)	5 (83%)	
PRRT		9 (32%)	9 (43%)		
TEM/CAP		2 (7%)	2 (10%)		
Other^b^		8 (29%)	6 (29%)	1 (17%)	
Overall best response to second-line treatment	27				
PR		4 (15%)	2 (10%)	2 (33%)	
SD		9 (33%)	8 (40%)	1 (17%)	
PD		6 (22%)	4 (20%)	1 (17%)	
NE/NA		8 (30%)	6 (30%)	2 (33%)	
mPFS second-line (95% CI)^a^	27	3.7 (2.8–4.6)	5.4 (0–11.6)	2.1 (0–4.6)	0.045
mOS second-line (95% CI)^a^	27	11.8 (0–25.1)	24.0 (4.0–44.0)	4.4 (1.3–7.5)	0.002

*NET* neuroendocrine tumour, *NEC* neuroendocrine carcinoma, *95% CI* 95% confidence interval, *PR* partial response, *SD* stable disease, *PD* progressive disease, *mDOR* median duration of response, *mPFS* median progression-free survival, *mOS* median overall survival, *PRRT* peptide receptor radionuclide therapy, *TEM* temozolomide, *CAP* capecitabine, *NE* not evaluable, *NA* not assessed/done.

^a^Months.

^b^Temozolomide monotherapy (*n* = 2), everolimus monotherapy (*n* = 2), streptozocin+5-FU (*n* = 2), capecitabine monotherapy (*n* = 1) and atezolizumab (*n* = 1).

^c^Breslow-test.

### [18F]FDG-PET/CT evaluation

Response evaluation with [18F]FDG-PET/CT after 6 weeks of treatment was available for 29 patients. Fifteen patients (52%) had a partial metabolic response, nine (31%) had stable metabolic disease, four (14%) had progressive metabolic disease, and one (3%) had a mixed metabolic response. Fourteen/29 patients (48%) had a non-concordant response according to PERCIST vs. RECIST (response was upgraded in nine patients by the PERCIST criteria). Patients with partial metabolic response had PFS 12.6 months (95% CI: 7.7–17.5 months) and OS 31.4 months (95% CI: 9.4–53.5 months). For patients with stable metabolic response and progressive metabolic disease, PFS was 8 months (95% CI: 5.2–10.8 months) and 3.3 months (95% CI: 0–11.8 months), and OS 13.9 months (95% CI: 0–31.2 months) and 23.1 months, respectively. Tumour-to-liver SUV_max_ uptake ratio at baseline was calculated and available for 21 patients. An SUV_TLR_ ≥ 3 showed a tendency towards shorter PFS (*p* = 0.058) but did not have any impact on OS.

### Univariate analysis

For the cohort as a whole, univariate analysis showed that Ki-67 > 55% (re-evaluated) and CRP > 10 mg/L were associated with shorter PFS (Table 3). Likewise, shorter OS was associated with performance status 1, Ki-67 > 55%, and CRP > 10 mg/L. A pancreatic primary did not affect PFS or OS.

**Table 3:** Cox regression on prognostic baseline factors for progression-free survival and overall survival.

	All (*n* = 37)		NET G3 *(n* = 26*)*	
Progression-free survival				
	**Hazard ratio (95% CI)**	***p*-value**	**Hazard ratio (95% CI)**	***p*-value**
Variables				
Performance status 0 vs. 1	1.6 (0.8–3.1)	0.196	1.3 (0.6–2.8)	0.582
Pancreatic primary vs. non-pancreatic primary	1.0 (0.5–2.0)	0.990	1.0 (0.4–2.3)	0.990
Partial response vs. stable disease	2.0 (1.0–4.4)	0.065	1.8 (0.7–4.6)	0.190
Ki-67 ≤ 55 % vs. >55 %	5.1 (2.0–13.2)	0.001	13.2 (2.2–79.9)	0.005
SRI uptake <liver vs. >liver	0.8 (0.3–2.1)	0.689	0.2 (0.0–0.8)	0.026
LDH ≤ UNL vs. >UNL	1.3 (0.6–2.8)	0.452	0.6 (0.2–1.7)	0.354
ALP ≤ UNL vs. >UNL	1.2 (0.6–2.5)	0.552	0.9 (0.3–2.2)	0.821
CRP ≤ 10 vs. >10	2.6 (1.2–5.5)	0.013	2.9 (1.1–7.2)	0.026
	**All**		**NET G3**	
Overall survival				
	**Hazard ratio (95% CI)**	***p*-value**	**Hazard ratio (95% CI)**	***p*-value**
Variables				
Performance status 0 vs. 1	2.3 (1.1–5.0)	0.034	2.3 (0.9–6.1)	0.099
Pancreatic primary vs. non-pancreatic primary	0.6 (0.3–1.3)	0.173	0.5 (0.2–1.2)	0.105
Partial response vs. stable disease	1.7 (0.7–4.1)	0.256	1.8 (0.6–5.7)	0.321
Ki-67 ≤ 55 % vs. >55 %	9.7 (3.1–30.7)	<0.01	38.2 (3.4–432.2)	0.003
SRI uptake <liver vs. >liver	0.8 (0.3–2.3)	0.690	0.8 (0.2–3.7)	0.783
LDH ≤ UNL vs. >UNL	1.2 (0.5–2.9)	0.659	0.5 (0.1–1.9)	0.343
ALP ≤ UNL vs. >UNL	1.7 (0.8–4.0)	0.198	1.7 (0.6–5.2)	0.333
CRP ≤ 10 vs. >10	2.2 (1.0–4.9)	0.047	2.1 (0.–5.8)	0.145

*SRI* somatostatin receptor imaging, *LDH* lactate dehydrogenase, *UNL* upper normal limit, *ALP* alkaline phosphatase, *CRP* C-reactive protein.

### Safety

**Fig. 2:**
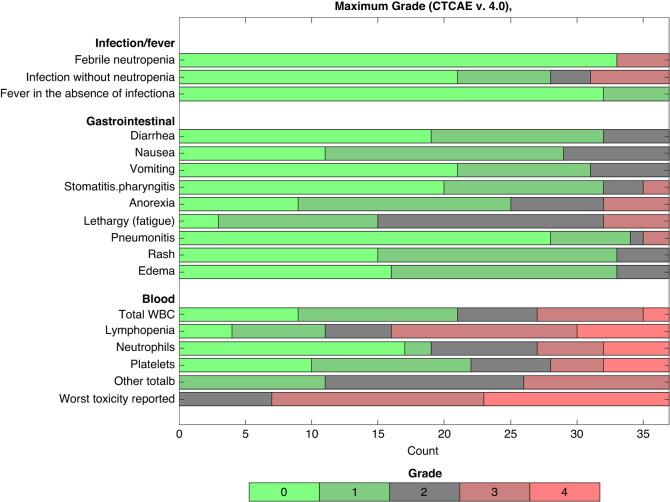
Toxicity reported under first-line treatment with everolimus/temozolomide. The stacked bar chart showing the safety profile according to Common Terminology Criteria for Adverse Events (CTCAE) version 4.0.

During study treatment, 43% experienced Grade 3 and 38% Grade 4 toxicity (Fig. 2). The most frequent Grade 3 adverse events were infection without neutropenia (16%), neutropenia (14%), anorexia (14%), fatigue (14%), febrile neutropenia (11%), and thrombocytopenia (11%) (Supplementary Table 1). Neutropenia and thrombocytopenia also commonly occurred as Grade 4 adverse events (14% each). Pneumonitis occurred in 24% of the patients, but only two patients (5%) experienced Grade 3 pneumonitis. Twenty-six patients (70%) experienced dose-limiting treatment-related toxicity, equally distributed between haematotoxicity and non-haematological toxicity. Twenty-two patients needed a dose reduction of both everolimus and temozolomide, and four patients needed a dose reduction of everolimus only. Twenty-three patients (62%) required dose delays (haematotoxicity: nine (39%), non-haematological toxicity: five (22%), both: eight (35%)). One case of GI bleeding due to thrombocytopenia was reported, which led to treatment discontinuation.

### Quality of life

**Fig. 3:**
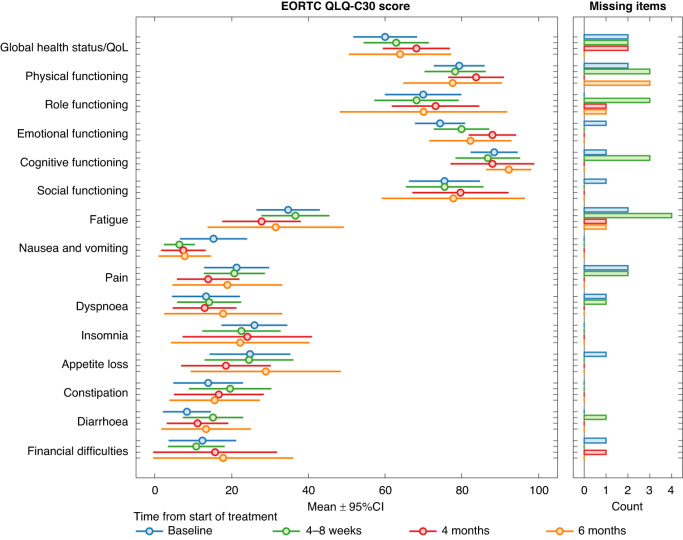
Quality of life (QoL) under first-line treatment with everolimus/temozolomide. Forest-plot describing changes in the QoL according to the European Organization for Research and Treatment of Cancer QoL questionnaire (EORTC QLQ-C30).

Quality of life was generally preserved during study treatment, with minor changes in the QoL mean score between baseline, 4 months (mean difference 8) and 6 months (mean difference 4) (Fig. 3).

### Second-line treatment

Twenty-eight patients received second-line treatment (76%), mainly PRRT (32%), carboplatin-etoposide (32%) or capecitabine/temozolomide (CAPTEM) (7%). RR was 15%, DCR 48%, and 22% experienced immediate PD. PFS and OS from the start of second-line treatment was 3.7 months (95% CI: 2.8–4.6 months) and 11.8 months (95% CI: 0–25 .1 months). Eleven and four patients received third- and fourth-line treatment.

### Subgroup analysis after pathological re-evaluation: NET G3 and NEC

All 37 patients were re-evaluated according to the 2019 WHO classification, identifying 26 NET G3, nine NEC, one mixed neuroendocrine-non-neuroendocrine neoplasm (MiNEN) and one case with ambiguous morphology. Pathological re-evaluation confirmed a Ki-67 ≤ 55% in 30 patients, while seven patients had Ki-67 > 55%, among them 4 NEC, 2 NET G3 and one case of MiNEN. Somatostatin receptor imaging (SRI) was positive in 17/20 NET G3 (85%) and 3/7 NEC patients (43%). Three NET G3 patients had a history of previous low/intermediate NET. Two patients experienced a transformation from NET to a tumour with NEC-like features, one before and one after study inclusion.

DCR at 6 months was 77% for NET G3 and 22% for NEC (*p* = 0.006). PR was achieved by seven patients (27%) with NET G3 and three patients (33%) with NEC. Median DOR for NET G3 and NEC was respectively 13.2 months (range 6.9–23.0 months) and 1.5 months (range 1.4–63.2 months). Three patients (8%) experienced immediate PD (two radiological and one clinical PD), two NEC and one NET G3, all with Ki-67 ≥ 70%. PFS for NET G3 was 12.6 months (95% CI: 8.7–16.5 months) vs. 3.4 months (95% CI: 2.0–4.7 months) for NEC (Log-rank-test: *p* = 0.133, Breslow-test: *p* < 0.01*)* (Fig. 1b). OS for NET G3 was 31.4 months (95% CI: 17.0–45.9 months) compared to 7.8 months (95% CI: 3.2–12.4 months) for NEC *(p* = 0.003*)* (Fig. 1c). Three-year survival was reached by 11 (42%) patients with NET G3 and one patient with NEC, all of which had a Ki-67 ≤ 55%. Four patients had a survival beyond 5 years, one NEC (with secondary surgery/ablation of metastatic disease) and three NET G3.

Univariate analysis for the NET G3 subgroup identified Ki-67 > 55% and CRP > 10 mg/L as predictors for shorter PFS, but not for OS. For the seven patients with Ki-67 > 55%, three patients had CRP ≤ 10, and four patients had CRP > 10.

### Second-line treatment

Second-line treatment was received by 21 patients with NET G3 (nine received PRRT) and six patients with NEC. After start of second-line treatment, NET G3 had a PFS of 5.4 months (95% CI: 0–11.6 months) compared to 2.1 months (95% CI: 0–4.6 months) for NEC (*p* = 0.045). OS after start of second-line treatment was 24 months (95% CI: 4–44 months) for NET G3 and 4.4 months (95% CI: 1.3–7.5 months) for NEC *(p* = 0.002).

## Discussion

To the best of our knowledge, this is the first prospective clinical trial examining everolimus and temozolomide as first-line treatment in metastatic HG GEP-NEN. The study’s primary endpoint was met (DCR at 6 months of 65%), PFS was 10.2 months and OS was 26.4 months. Grade 3–4 toxicity was commonly observed. Dose reductions, dose delay and treatment discontinuation due to toxicity were registered in 26 (70%), 23 (62%), five (14%) patients, respectively, but the quality of life remained stable through the treatment period.

The intention of the study was to include high-grade GEP-NEN with a Ki-67 ≤55%. Due to the changing WHO classification, a re-evaluation of morphology and Ki-67 index was performed to separate NET G3 from NEC. NET G3 and NEC are two distinct disease entities, supported by their different responses to platinum-based chemotherapy, survival, and molecular profile [4, 6, 27]. Limited data exist on NET G3; previous studies have reported that they constitute 12-18% of all HG GEP-NEN [4, 6, 27]. In our material, as many as 70% were re-classified as NET G3, most likely caused by the Ki-67 ≤ 55% inclusion criterion. Seven patients had a Ki-67 > 55% after re-evaluation. To our surprise, very few GEP-NEC patients had a Ki-67 < 55%, indicating that this subgroup might be small [4]. Like earlier reports, most NET G3 patients had a pancreatic primary, and the median Ki-67 was 29% [4, 6, 28].

There are limited data on first-line treatment for advanced GEP-NET G3 and mainly retrospective data are available [15–17, 29] (Supplementary Table 2). Platinum-based chemotherapy is recognised as a poor treatment option for most GEP-NET G3 patients [6, 30, 31] and is generally not recommended as a first-line treatment in current guidelines [15–18].The choice of treatment for these patients may depend on clinical judgement considering disease burden, proliferation index, rate of disease progression, and SRI avidity.

Comparing our results to previous studies on systemic treatment for GEP-NET G3 are difficult, as these studies include retrospective data, a mixture of different treatment lines, inclusion of non-digestive primaries, and lack of pathological review of HG GEP-NEN according to the 2019 WHO classification. Studies on first-line treatment with platinum/etoposide in NET G3 show a PFS of 5.0–6.9 months [6, 32]. Better efficacy outcomes have been reported for CAPTEM (RR 23–41%, PFS 5.7–14.1 months and OS 31.7–41.2 months) [7–9, 33, 34] and FOLFOX (RR 25-56% and PFS 6.9-16.5 months) [32, 33]. Retrospective data supports FOLFOX as a therapeutic option for advanced GEP-NEN G3 [35]. The combination of temozolomide and nivolumab in patients with NEN, showed a RR 32.1%, PFS 8.8 months and OS 32.3 months, regardless of tumour differentiation and proliferation rate [36]. In our first-line study, we found for NET G3 a RR at 27%, PFS 12.6 months and OS 31.4 months, comparable to prior study results on NET G3. Interestingly, we found no differences in benefit for pancreatic vs. non-pancreatic NET G3 patients.

Our findings indicate that everolimus/temozolomide may be considered a treatment option for selected GEP-NET G3 patients. The combination might be an alternative especially for patients with significant cardiovascular disease or a complete lack of dihydropyrimidine dehydrogenase [37], avoiding capecitabine (CAPTEM) and 5-FU (FOLFOX). Like CAPTEM, it offers the advantages of being an oral regime.

The optimal way to treat GEP-NEC patients with a Ki-67 < 55% is uncertain, with published studies indicating a poorer response to platinum/etoposide [4, 5, 8]. GEP-NEC patients with a Ki-67 < 55% showed a RR of 25%, PFS of 5 months and OS of 11 months, after receiving platinum/etoposide treatment [6]. A small prospective phase II study evaluating CAPTEM in NEN G3 with Ki-67 < 55% showed that the NEC subgroup did not improve their outcome compared to historical data on platinum/etoposide [34]. A first-line study of CAPTEM vs. platinum/etoposide in non-small cell digestive NEC and NET G3 was closed early due to poor accrual [38]. Among included 62 evaluable patients, RR, PFS and OS with CAPTEM were 9%, 2.4 months and 12.6 months vs. 10%, 5.4 months and 13.6 months with platinum/etoposide. The study interpretation is limited by including both NET G3 and NEC patients. In our study, PFS and OS (3.4 months and 7.8 months) by first-line everolimus/temozolomide, suggest a limited benefit of this schedule in GEP-NEC although only nine patients were included. The OS for the NEC subgroup seems inferior to the 11–12 m OS seen in prior GEP-NEC studies with the use of first-line platinum/etoposide [10–13]. Our study verifies the significant survival advantage of patients with metastatic GEP-NET G3 in comparison to NEC (31.4 months vs. 7.8 months, *p* = 0.003), like prior observations by Elvebakken et al. (33 months vs. 11 months) [6].

Elevated CRP at baseline was a significant poor prognostic factor for PFS and OS among all patients and for PFS in the NET G3 subgroup. CRP is a well-known prognostic factor for other cancers and for survival in surgically resected pancreatic G1-G3 NEN [39]. Future GEP-NEN studies should further explore CRP as a possible predictive and prognostic marker.

[18F]FDG-PET is known to show a high uptake in GEP-NEC and in the majority of NET G3 patients [4, 12, 40]. In our cohort, all 33 patients with [18 F]FDG-PET showed avidity. According to PERCIST, partial metabolic responders had a better OS than patients with stable metabolic disease. In our study, RECIST seemed to better correlate PR with longer PFS than PERCIST (PFS 18.3 months vs. 12.6 months). PERCIST upgraded nine patients from SD to PR, and PERCIST appeared to better discriminate survival comparing PR vs.SD (OS 31.4 months vs. 13.9 months compared to 29.7 months vs. 25.1 months with RECIST). Surprisingly the four patients with progressive metabolic disease showed a longer OS than those with stable metabolic disease. More data are needed to understand the possible role of PERCIST in treatment evaluation. SUV_TLR_ of 3 at baseline showed a trend towards shorter PFS, in line with previous reports, which suggests a correlation between poor prognosis and increased FDG uptake in NEN [41]. The majority of our patients had an SRI uptake ≥liver as in prior studies [42]. Retrospective studies have shown that PRRT might be a treatment option for GEP-NET G3 and possibly for selected GEP-NEC patients with Ki-67 ≤ 55% [43–45].

The safety profile in our study was consistent with previous reports on everolimus/temozolomide, but with a high number of patients experiencing grade 3–4 toxicity (81%), and the majority needing dose reduction (70%) and/or treatment delay (62%). The rate of haematological toxicity (neutropenia grade 3–4 28% vs. 7% and trombocyopenia grade 3–4 25% vs. 16%) and all grades of pneumonitis (24 % vs. 7%) was higher in our study compared to the prospective data on everolimus/temozolomide provided by Chan et al. [23]. However, discontinuation due to adverse events was lower in our study, 14 vs. 21%. Temozolomide 150 mg/m^2^ every 7 days every other week was used in our study, but the optimal starting dose for temozolomide is unknown. Other studies have reported a good tolerance and response with 5 days of temozolomide 200 mg/m^2^ every 4 weeks [7, 46]. The optimal starting dose for temozolomide and the question of continuing maintenance everolimus after a fixed number of temozolomide cycles is yet to be explored in future studies. A starting dose of temozolomide at 100 mg/m^2^ every other week (equivalent to -1 dose level in this study) might be reasonable. Comparing the safety profile against both prospective and retrospective data (where toxicity evaluation is not standardised) on CAPTEM gives the impression that CAPTEM is generally better tolerated than everolimus/temozolomide with less grade 3–4 toxicity. Although discontinuation due to treatment-related toxicity has been reported between 6-16 % for CAPTEM [7, 34, 47, 48]. Even if Grade 3–4 toxicity was frequent in our study, self-reported QoL remained stable from the initiation of treatment and through the following 4–6 months.

### Strengths and limitations

The prospective design of the study and re-evaluation of HE and immunohistochemically stained slides (including Ki-67) by experienced NEN pathologists according to the 2019 WHO classification are major strengths of this study. The main limitations are the trial’s single-arm design, which makes it difficult to compare the results to other treatment regimes, and the limited number of patients due to the infrequency of these neoplasms. Interpretation of the statistical analyses should therefore be done with caution. Further studies are warranted to decide if first-line everolimus and temozolomde may be recommended treatment options for GEP-NET G3. A larger randomised study with CAPTEM as a comparator would have more impact but is, unfortunately, most likely not feasible. The use of everolimus in a first-line treatment regime could possibly impair the use of this drug in second- or third-line settings for NET G3. The recent changes in HG GEP-NEN nomenclature were not in place at the study’s initiation; therefore, this is a combined NET G3 and NEC cohort. After a histopathology review, seven patients had a Ki-67 > 55%. These patients were included in the final analysis, which seems reasonable considering the unknown optimal cut-off for Ki-67 and the interpersonal variability in Ki-67 reporting. Adequate pathological assessment will be essential in future clinical trials to further assess optimal therapeutic options for NET G3 and NEC. Our survival results may be influenced by the selection of patients with performance status ≤1. In this study, the O-6-Methylguanine-DNA Methyltransferase (MGMT) methylation status was not analysed, and NGS analyses are lacking for most patients. Studies have shown conflicting results on the predictive value of MGMT for the benefit of temozolomide treatment; however, a recent study found a correlation to RR for pancreatic NET G1-2 patients [8, 34, 48–50].

## Conclusion

In summary, everolimus and temozolomide may be a treatment option for selected patients with metastatic GEP-NET G3 resulting in a high DCR at 6 months of 77%, PFS 12.6 months, and OS 31.4 months. The benefit for the few GEP-NEC patients was limited. Careful monitoring of toxicity is essential when using this combination, but toxicity did not compromise QoL.

### Supplementary information

Supplementary information

Supplementary information

## Data Availability

The data generated and analysed in this study are available from the corresponding author upon reasonable request.
