# Supplementary material for: Phase II study of everolimus and temozolomide as first-line treatment in metastatic high-grade gastroenteropancreatic neuroendocrine neoplasms
Source: Br J Cancer. 2023 Oct 23;129(12):1930–9. doi: 10.1038/s41416-023-02462-0 (PMC10703888; doi:10.1038/s41416-023-02462-0)
Supplement: Supplementary file 1 — Supplementary information [file 41416_2023_2462_MOESM1_ESM.docx]

**Supplementary Table 1.** Adverse events (CTCAE v. 4.0) during first-line treatment with everolimus and temozolomide in 37 patients with advanced high-grade gastroenteropancreatic neuroendocrine neoplasms

| **Adverse Event** |  | | | | |
| --- | --- | --- | --- | --- | --- |
|  |  | Grade 1 | Grade 2 | Grade 3 | Grade 4 |
| Infection/fever |  |  |  |  |  |
| Febrile neutropenia | 37 | - | - | 4 (11%) | 0 |
| Infection without neutropenia | 37 | 7 (19%) | 3 (8%) | 6 (16%) | 0 |
| Fever in the absence of infection^a^ | 37 | 5 (14%) | 0 | 0 | 0 |
| Gastrointestinal |  |  |  |  |  |
| Diarrhoea | 37 | 13 (35%) | 5 (14%) | 0 | 0 |
| Nausea | 37 | 18 (49%) | 8 (22%) | 0 | 0 |
| Vomiting | 37 | 10 (27%) | 6 (16%) | 0 | 0 |
| Stomatitis/pharyngitis | 37 | 12 (33%) | 3 (8%) | 2 (5%) | 0 |
| Anorexia | 37 | 16 (43%) | 7 (19%) | 5 (14%) | 0 |
| Lethargy (fatigue) | 37 | 12 (32%) | 17 (46%) | 5 (14%) | 0 |
| Pneumonitis | 37 | 6 (16%) | 1 (3%) | 2 (5%) | 0 |
| Rash | 37 | 18 (49%) | 4 (11%) | 0 | 0 |
| Oedema | 37 | 17 (46%) | 4 (11%) | 0 | 0 |
| Blood |  |  |  |  |  |
| Total WBC | 37 | 12 (33%) | 6 (16%) | 8 (22%) | 2 (5%) |
| Lymphopenia | 37 | 7 (19%) | 5 (14%) | 14 (38%) | 7 (19%) |
| Neutrophils | 37 | 2 (5%) | 8 (21%) | 5 (14%) | 5 (14%) |
| Platelets | 37 | 12 (32%) | 6 (16%) | 4 (11%) | 5 (14%) |
| Other total^b^ | 37 | 11 (30%) | 15 (40%) | 11 (30%) | 0 |
| Worst toxicity reported | 37 | 0 | 7 (19%) | 16 (43%) | 14 (38%) |

^a^Included drug fever

^b^ Other reported grade 1 - 2 toxicity with >10% incidence : constipation (38%), weight loss (24%), anaemia (19%), pain (19%), dyspnoea (16%), other skin toxicity (16%), increased liver enzymes (14%), hyperglycaemia (14%), dyslipidaemia (14%), pruritus (11%), cough (11%)

Abbreviations: CTCAE, Common Terminology Criteria for Adverse Events; WBC, white blood cells
