# Supplementary material for: Phase II study of everolimus and temozolomide as first-line treatment in metastatic high-grade gastroenteropancreatic neuroendocrine neoplasms
Source: Br J Cancer. 2023 Oct 23;129(12):1930–9. doi: 10.1038/s41416-023-02462-0 (PMC10703888; doi:10.1038/s41416-023-02462-0)
Supplement: Supplementary file 2 — Supplementary information [file 41416_2023_2462_MOESM2_ESM.docx]

**Supplementary Table 2.** Study results on the use of temozolomide-based chemotherapy for metastatic gastroenteropancreatic neuroendocrine tumours grade 3 (NET G3)

|  |  | Line | n | Treatment | RR | PFS | OS |
| --- | --- | --- | --- | --- | --- | --- | --- |
| Spada F:  Endocrine 2020,  72, 268-78 | NET G3 | 78% pre-treated | 40 | CAPTEM and TEM | 23% | 14.1 m | 35.6 m |
| Liu AJ:  Oncologist 2021, 26, 383-388 | NET G3 | 50% 1-line | 20 | CAPTEM | 35% | 9.4 m | 41.2 m |
| Chan DL:  Oncologist 2021, 26, 950-955 | NET G3 | Mixed | 64 | CAPTEM | 41% | 5.7 m | 31.7 m |
| De Mestier L:  ERC 2021, 28, 549-561 | NET G3 | 1/2 -line | 32  20  22  21 | Alkylating (temozolomide)  AdC (FOLFOX)  Etoposide/platinum  SSA | 38%  25%  12%  0 | 7.8 m  16.5 m  7.2 m  6.2 m | 36 m (from 1-line) |
| Apostolidis L:  Cancers 2021, 13, 1936 | NET G3 | 1-line | 37  39  22  20 | Etoposide/platinum  FOLFOX  CAPTEM  SZT/5-FU | 35%  56%  27%  45% | 6.9 m  6.9 m  12 m  4.8 m |  |
| Jeong H:  ESMO Open 2021, 6(3), 100119 | NET G3 | Mixed | 23 | CAPTEM | 35% | 9.3 m | Not reached |
| Morken 2023 | NET G3 | 1-line | 26 | Temozolomide+  everolimus | 27% | 12.6 m | 31.4 m |

Abbreviations: CAPTEM, capecitabine amd temozolomide; TEM, temozolomide; m, months; AdC, adenocarsinoma-like chemotherapy; SSA, somatostatin analogue; SZT/5-FU, streptozotocin/5-fluorouracil
